# Atorvastatin Treatment Does Not Alter Pulse Wave Velocity in Healthy Adults

**DOI:** 10.1155/2014/239575

**Published:** 2014-11-13

**Authors:** Kevin D. Ballard, Beth A. Taylor, Jeffrey A. Capizzi, Adam S. Grimaldi, C. Michael White, Paul D. Thompson

**Affiliations:** ^1^Division of Cardiology, Henry Low Heart Center, Hartford Hospital, Hartford, CT 06102, USA; ^2^Department of Health Sciences and Nursing, University of Hartford, West Hartford, CT 06117, USA; ^3^School of Pharmacy, University of Connecticut, Storrs, CT 06269, USA

## Abstract

*Introduction*. Both statins and regular physical activity (PA) reduce arterial stiffness. The present post hoc analysis examined if arterial stiffness was improved with high-dose atorvastatin treatment in healthy adults and whether PA levels magnified this response. We utilized data from a double-blind, random-assignment clinical trial investigating the effects of atorvastatin 80 mg/d for 6 mo on skeletal muscle symptoms. *Methods*. Central and peripheral arterial pulse wave velocity (PWV) were measured and PA levels assessed at baseline and 6 mo in subjects randomized to atorvastatin (*n* = 21, 9 men) or placebo (*n* = 29, 16 men). *Results*. Baseline participant characteristics, PWV, and PA levels were not different between treatments. Central (means ± SD; 8.7 ± 2.6 to 9.0 ± 2.5 m/sec) and peripheral PWV (9.9 ± 1.3 to 9.8 ± 1.6 m/sec) were unchanged from baseline following atorvastatin treatment (time × drug interaction: *P* ≥ 0.13). Similarly, PA levels were unaffected by time or treatment. In sex and age adjusted models, baseline levels of PA were not related to changes in PWV with atorvastatin treatment. *Conclusion*. These data indicate that high-dose atorvastatin treatment for 6 mo does not influence arterial stiffness in healthy adults. Participation in habitual PA did not magnify the vascular effects of statin therapy. This study was registered with ClinicalTrials.gov NCT00609063.

## 1. Introduction

Beyond effective lowering of low-density lipoprotein cholesterol (LDL-C) concentrations, hydroxymethylglutaryl coenzyme A (HMG-CoA) reductase inhibitors (statins) are associated with multiple vascular benefits [1, 2] that may contribute to reduced cardiovascular disease (CVD) morbidity and mortality [3–6]. One such vascular benefit is a reduction in central arterial stiffness (assessed noninvasively by arterial pulse wave velocity (PWV)) that has been reported in some [7–11] but not all [12–14] studies. Reductions in central arterial stiffness with statin use have been observed in clinical populations characterized by increased CVD risk, including hypertension [7], excess body mass [10], and hypercholesterolemia [11], as well as healthy middle-aged men [8]. Additionally, regular PA is associated with a reduction in central arterial stiffness [15–17]. Specifically, central PWV was inversely related to the daily time spent performing moderate-intensity PA evaluated by accelerometry in 403 adults >40 y of age [15], suggesting that regular PA may exert destiffening effects. Statin-mediated improvement of arterial stiffness in healthy adults and its relationship with PA remain unclear.

The present investigation examined if PWV was improved in a sample of participants from the larger STOMP (Effect of Statins on Muscle Performance) study (NCT00609063), of which the methods have been described in detail [18, 19]. Additionally, we determined if habitual PA levels magnified the vascular effect of statin therapy. We hypothesized that atorvastatin treatment would reduce arterial PWV and that this beneficial vascular effect would be enhanced in physically active adults.

## 2. Materials and Methods

### 2.1. Study Design

STOMP was a double-blind, randomized controlled trial (RCT) investigating the effects of 6 mo treatment with atorvastatin 80 mg/d or placebo on skeletal muscle performance and muscle symptoms in 419 healthy statin-naïve adults (aged 20–76 y) [18]. In the present study, serological markers, PA levels, and arterial stiffness parameters were measured at baseline and following 6 mo of daily treatment with atorvastatin 80 mg (*n* = 21 of 202) or placebo (*n* = 29 of 217). The Institutional Review Boards at Hartford Hospital, University of Massachusetts, and University of Connecticut approved the study and the study was monitored by a Data Safety and Monitoring Board.

### 2.2. Physical Activity Assessment

Subjective and objective assessment of PA levels occurred at baseline and 6 mo using the Paffenbarger PA Questionnaire and accelerometers, respectively, as described [18, 19]. Specifically, participants' habitual PA levels were assessed using the Paffenbarger PA Questionnaire [20], a well-established tool designed for population measurement of leisure-time physical activity. Additionally, participants' PA levels were objectively measured using an Actigraph (Actigraph, Pensacola, FL) accelerometer recorder over a 96 h period.

### 2.3. Arterial Stiffness Assessment

Measurement of arterial stiffness parameters occurred at Hartford Hospital. Following a 10 min supine rest period, measurements of PWV and pulse wave analysis were performed with the SphygmoCor CPV Central Blood Pressure/Pulse Wave Velocity System (AtCor Medical, Sydney, Australia). Multiple pulse waveforms of the right carotid and right femoral artery were recorded sequentially by applanation tonometry to determine central PWV. The aortic transit time (*T*_*R*_) was determined by measuring the distance between the points of measurement of the carotid and femoral pulses, recorded by taking measurements on the surface of the body from the suprasternal notch to the point where the right carotid pulse was found and from the suprasternal notch to the right femoral pulse via the umbilicus. Peripheral PWV was measured as the transit time between the right radial and the right femoral artery waveforms. Pulse waveforms obtained over a 10 sec period at the right radial artery were used to compute a corresponding central waveform using a validated mathematical transformation. Augmentation index (AIx) with and without standardization to a heart rate of 75 bpm (AIx at HR_75 bpm_) is reported as pulse wave analysis parameters.

### 2.4. Statistical Analyses

A power calculation was performed (PS Power and Sample Size Calculation version 3.0.43; Vanderbilt University, Nashville, TN, USA) utilizing data from a previous parallel design study examining changes in PWV following treatment for 6 mo with atorvastatin 10 mg/d or placebo [7]. With 21 subjects per group, the present study had >80% power to detect a difference of 1.9 m/sec in central PWV between atorvastatin- and placebo-treated subjects at 6 mo, with *α* = 0.05 and a standard deviation of 2.1 m/sec. Data (means ± SD) were analyzed by SPSS Version 19.0 (SPSS Inc., Chicago, IL, USA). Data were assessed for normality using Shapiro-Wilk's* W*-test prior to all analyses. No differences were observed between atorvastatin-treated subjects who did (*n* = 4) or did not (*n* = 17) meet the study definition for myalgia [18, 19]. Therefore, all subjects were included in the analyses. Baseline values in atorvastatin- and placebo-treated subjects were evaluated using one-way ANOVA, a Mann-Whitney* U* test, or a Pearson chi-square test. Change scores (post-pre) for serum lipids were assessed with independent samples* t*-tests comparing atorvastatin- and placebo-treated subjects. Two-way repeated measures ANOVA was used to determine differences due to treatment, time, and their interaction for arterial stiffness measures and PA levels. Linear regression was performed to evaluate if baseline values or changes in study variables predicted changes in arterial stiffness, with further models controlling for sex and age. An *α*-level of *P* ≤ 0.05 was considered statistically significant for all analyses.

## 3. Results

Characteristics of participants who completed the intervention are summarized in Table 1 and were not different between treatments (all *P* ≥ 0.08).

**Figure 1:**
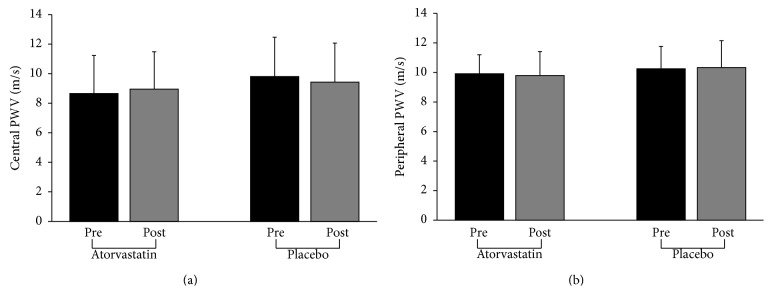
Central (a) and peripheral (b) pulse wave velocity (PWV) before (Pre) and after (Post) 6 months of atorvastatin (*n* = 21) or placebo (*n* = 29) treatment. Data are means ± SD.

Atorvastatin treatment produced the expected reductions in serum lipids (Table 2). Central and peripheral PWV measures did not differ between groups at baseline (*P* ≥ 0.13) and no changes were observed between baseline and study end within a treatment group (time × drug interaction: *P* ≥ 0.23) (Figure 1). No differences were observed between or within groups for *T*_*R*_, AIx, or AIx at HR_75 bpm_ (*P* ≥ 0.13) (Table 3). Changes in serum lipids with atorvastatin treatment were not related to changes in arterial stiffness (all *P* ≥ 0.16).

Baseline PA levels did not differ between treatment groups (Table 1) and no changes were observed for PA levels within a treatment group (time × drug interaction: *P* ≥ 0.09). In the atorvastatin group only, baseline levels of moderate-intensity PA were inversely related (*r* = −0.55, *R* ^2^ = 0.30, *P* = 0.02) to the change in peripheral PWV suggesting that high levels of moderate PA magnify the arterial destiffening effect of statins. In contrast, baseline levels of vigorous PA were directly related (*r* = 0.56, *R* ^2^ = 0.32, *P* = 0.02) to changes in peripheral PWV with atorvastatin treatment. These relationships were no longer significant after adjustment for sex and age (*P* ≥ 0.09).

## 4. Discussion

Prospective data indicate that increased aortic PWV independently predicts CVD and all-cause mortality [21]. Central arterial destiffening with statin therapy was observed in patients at increased CVD risk [7, 10, 11], whereas others have found no effects in various clinical populations [12–14]. Existing evidence from RCTs is contradictory regarding a reduction in arterial stiffness with statin therapy [22]. In the present study, 80 mg/d atorvastatin for 6 mo had no effect on central and peripheral PWV in healthy adults from STOMP [18].

Statin-mediated reductions in vascular smooth muscle tone via improvements in endothelial function [1] or suppression of sympathetic neural activity [23, 24] have been proposed as mechanisms by which statins reduce central arterial stiffness in humans. The present placebo-controlled study documents that high-dose atorvastatin therapy does not impact arterial stiffness parameters in clinically asymptomatic adults. In contrast, utilizing a parallel study design Lunder et al. [8] found that central PWV measured by ultrasonography decreased (*P* < 0.001) by 6.2% from baseline, indicative of improved arterial stiffness, in 25 healthy men (43.5 ± 1.6 y) randomized to fluvastatin 10 mg/d for 30 d. Central PWV was unchanged in the placebo group (*n* = 25). In these same subjects, endothelium-dependent brachial artery flow-mediated dilation increased by 92% within 30 d (2.4 ± 0.3% to 4.4 ± 0.3%; *P* < 0.001), suggesting that low-dose fluvastatin rapidly improved both functional and morphological arterial measures. Improvements in vascular function were independent of lipid lowering as plasma lipid concentrations remained unchanged with fluvastatin [8]. Serum LDL-C concentrations decreased by 51% following 6 mo atorvastatin treatment in our small cohort of subjects from STOMP [18], an effect not associated with changes in arterial stiffness. Intrinsic differential effects of statins on arterial stiffness [25] and/or heterogeneous protocols for PWV assessment [22] may explain the discrepant results of the present study conducted in healthy adults free of CVD.

Habitual PA is an effective lifestyle intervention to decrease central arterial stiffness [15–17], thus lowering CVD risk. In the present study we investigated if subjective and objective measures of PA magnified the arterial destiffening effect of statins. In unadjusted models we observed that baseline levels of moderate-intensity PA were inversely associated with a greater reduction in peripheral PWV with atorvastatin, suggesting that statin-mediated vascular benefits may be enhanced in physically active adults. However, this relationship no longer persisted following adjustment for sex and age. Conversely, levels of vigorous PA assessed at baseline were directly related to an increase in peripheral PWV with 6 mo atorvastatin therapy in unadjusted models. As only 7 of 21 atorvastatin-treated subjects participated in vigorous PA, and fewer accumulated >10 min/d (2 of 21), more research is needed to determine if the combination of statins and vigorous PA negatively affects arterial stiffness. We recommend that PA levels be monitored in future RCTs to better determine the influence of regular participation in PA on statin-mediated vascular responses.

The current post hoc analysis of a sample of participants from STOMP [18] has some limitations that must be acknowledged. Our observation of unchanged arterial stiffness with high-dose atorvastatin treatment for 6 mo may be explained by the small number of subjects in each group. However, we were adequately powered to detect improvements in central PWV based on data from a 6 mo parallel design study examining the effects of low-dose (10 mg/d) atorvastatin or placebo (*n* = 25/group) in patients with hypertension and hypercholesterolemia [7]. Notably, the 18% decrease in central PWV observed with atorvastatin [7] can be considered clinically significant as a similar magnitude of improvement was observed in obese individuals [10] and hypercholesterolemic patients [11] following statin treatment. Participants in STOMP were rigorously screened at baseline to exclude those with a history of CVD or other factors that would confound study outcomes [18, 19]. Additionally, between study differences in statin type/dose, treatment duration, underlying comorbidities, and PWV protocols preclude clarification of the effect of statin therapy on arterial stiffness [22]. Future studies should determine if lower statin doses exert beneficial pleiotropic effects on vascular function, an effect observed previously in mice [26]. Additionally, future RCTs are warranted evaluating PWV as the primary study outcome by utilizing a standardized assessment protocol applied to a sufficient number of homogeneous subjects. Increasing statin use [27] and the positive association between increased central PWV and CVD mortality [21] necessitates further investigation to more definitively determine whether statins may be an effective vascular destiffening therapy.

## 5. Conclusion

Results from our RCT in a sample of healthy adults from STOMP [18] suggest that high-dose atorvastatin for 6 mo does not influence arterial stiffness. Our observation in unadjusted models of a reciprocal relationship between moderate versus vigorous PA and changes in arterial stiffness with statin use deserves clarification in a larger cohort.

## Figures and Tables

**Table 1:** Baseline characteristics of study participants by drug assignment.

	Atorvastatin (*n* = 21)	Placebo (*n* = 29)	*P* value
Age, y	52.6 ± 15.7	58.7 ± 8.1	0.08
Men, *n* (%)	9 (43%)	16 (55%)	0.39
Height, cm	170.7 ± 11.4	171.8 ± 8.8	0.69
Weight, kg	80.9 ± 18.4	78.3 ± 17.0	0.62
BMI, kg/m^2^	27.3 ± 4.0	26.4 ± 4.4	0.47
HR, bpm	67 ± 9	69 ± 11	0.49
SBP, mmHg	119.2 ± 13.7	115.4 ± 12.2	0.30
DBP, mmHg	75.5 ± 8.9	72.6 ± 7.8	0.23
TC, mmol/L	5.24 ± 1.26	5.36 ± 0.86	0.70
LDL-C, mmol/L	3.05 ± 1.17	3.27 ± 0.75	0.43
HDL-C, mmol/L	1.58 ± 0.54	1.62 ± 0.47	0.76
TG, mmol/L	1.34 ± 0.73	1.13 ± 0.47	0.22
CK, U/L	157.1 ± 110.4	120.8 ± 77.8	0.14
Activity counts, counts/d^a^	169.6 ± 160.8	166.5 ± 115.0	0.94
Moderate activity, min/d	103.7 ± 51.8	112.9 ± 50.0	0.58
Vigorous activity, min/d	6.5 ± 13.8	5.2 ± 9.6	0.74

Data are means ± SD or proportions. BMI, body mass index; C, cholesterol; CK, creatine kinase; DBP, diastolic blood pressure; HR, resting heart rate; LDL, low-density lipoprotein; HDL, high-density lipoprotein; SBP, systolic blood pressure; TG, triglycerides.

^a^In thousands.

**Table 2:** Serum lipid changes by drug assignment.

	Atorvastatin (*n* = 21)	Placebo (*n* = 29)
ΔTotal-C, mmol/L	−1.83 ± 0.93^*^	0.05 ± 0.50
ΔLDL-C, mmol/L	−1.65 ± 0.93^*^	0.01 ± 0.44
ΔHDL-C, mmol/L	−0.02 ± 0.24	−0.00 ± 0.20
ΔTG, mmol/L	−0.35 ± 0.68^*^	−0.01 ± 0.34

Data are means ± SD. Δ, absolute change from before to after the treatment intervention; C, cholesterol; LDL, low-density lipoprotein; HDL, high-density lipoprotein; TG, triglycerides.

^*^ *P* < 0.05 from baseline.

**Table 3:** Arterial stiffness changes by drug assignment.

	Atorvastatin (*n* = 21)		Placebo (*n* = 29)	
	Baseline	6 mo	Baseline	6 mo
*T*_*R*_, ms	139.1 ± 36.8	148.4 ± 18.8	147.7 ± 19.4	143.4 ± 18.0
AIx, %	21.5 ± 15.1	20.1 ± 14.3	24.8 ± 10.4	27.6 ± 14.3
AIx at HR_75 bpm_, %	16.5 ± 14.2	16.0 ± 15.2	18.4 ± 10.6	22.1 ± 14.4

Data are means ± SD. *T*_*R*_, aortic transit time; AIx, augmentation index; AIx at HR_75 bpm_, AIx normalized to heart rate of 75 bpm.
